# Seasonal Variation in the Diagnosis of Skin Cancers From 1983 to 2017 in Greenville, North Carolina

**DOI:** 10.7759/cureus.23254

**Published:** 2022-03-17

**Authors:** Arthur M Samia, Joseph Nenow

**Affiliations:** 1 Dermatology, University of Florida, Gainesville, USA; 2 Division of Medicine, Brody School of Medicine/East Carolina University, Greenville, USA

**Keywords:** non-melanoma skin cancer, medical dermatology, cutaneous oncology, melanoma skin cancer, clinical dermatology

## Abstract

Background

Seasonality of diagnosis occurs in many types of cancer and is well-established in non-melanoma (NMSC) and melanoma (MSC) skin cancers. Benign skin conditions have also been shown to demonstrate a similar seasonality pattern. Investigations into the seasonality of NMSC and MSC diagnoses are less common than benign skin conditions despite the high healthcare burden of the disease. In this study, we investigated if seasonality and monthly patterns of NMSC and MSC diagnoses are present in Eastern North Carolina.

Methodology

We observed and analyzed incident cancer diagnoses for patients visiting the Physicians East Dermatology clinic in Greenville, North Carolina, from 1983 to 2017 (n = 8,021 basal cell carcinomas (BCCs), n = 5,660 squamous cell carcinomas (SCCs), n = 451 MSCs, n = 14,132 total).

Results

Chi-square tests showed the highest rates of diagnosis for BCCs in August (9.85%), September (9.62%), and October (10.0%). For SCCs, the diagnosis rates were the highest in July (8.62%), August (9.63%), and October (9.58%). For MSCs, the diagnosis rates were the highest in May (9.98%), June (10.2%), and July (10.4%). Analysis of the differences between observed skin cancer diagnoses by month and equal distribution across all months in the event of no seasonality revealed peaks of skin cancer diagnoses corresponding to July through October for BCCs; July, August, and October for SCCs; and May through September for MSCs. Analysis of the patterns of diagnosis of this data over 34 years illustrated a continuously increasing pattern of diagnosis for all three cancer subtypes from 1983 to 2017.

Conclusions

This study identified a statistically significant pattern of seasonality in both NMSCs and MSCs, which was consistent with the findings of previous studies. Moving forward, further research should investigate the roles of temperature, quantified ultraviolet exposure, and geographic location and their relationships to seasonality.

## Introduction

Seasonality of diagnosis for non-melanoma (NMSCs) and melanoma (MSCs) skin cancers is well-established for individuals with fair skin types in various global geographies [1-3]. These trends in seasonality have significant implications for patient morbidity, mortality, and costs to the healthcare system [4]. A review of previous studies indicates seasonal trends are rooted in three factors, namely, seasonal variations in access to healthcare, inhibited skin examination due to clothing, and increased seasonal sun exposure [1,5,6]. While some combination of all three factors is likely, proponents of variation in access to care suggest that sun-awareness health initiatives often occur during the summer and encourage seasonal variation through the selective promotion of care, which may affect the frequency of patient self-evaluation as well as contribute to the observed seasonal variation in the diagnosis of benign skin lesions [6,7]. Exposure to sunlight has been suggested to affect the seasonality of diagnosis and prognosis through a vitamin D-dependent mechanism in many forms of cancer [8-10]. Short-term ultraviolet (UV) promotional effect on melanocytes has been suggested to lead to seasonal variations in diagnosing thin melanomas [11]. The goals of this study were to explore seasonality in relationship to both MSCs and NMSCs and discuss novel potential cofounders. Specifically, we hope to answer the following: (1) do these trends of seasonality exist for our patient population in Greenville, North Carolina from 1983 to 2017?; (2) if so, are the peak and trough months similar or different between MSCs and NMSCs?; and (3) what information would best allow a clearer understanding of the causes behind these trends in seasonality? Given these questions, our study is significant for its combination of length, dual exploration of NMSC and MSC subtypes, and the novel consideration of potential confounders in consideration of ultraviolet radiation (UVR) exposure.

## Materials and methods

Study design

We observed and analyzed incident NMSC and MSC diagnoses for patients visiting the Physicians East Dermatology clinic in Greenville, North Carolina, from 1983 to 2017. The institutional review board approved the study (UMCIRB 10-0534).

Inclusion and exclusion criteria

Only basal cell carcinomas (BCCs), squamous cell carcinomas (SCCs), and MSCs were included in this study. All other forms of skin cancer were excluded, but patients were not excluded for any other reason.

Data collection

Data regarding NMSCs and MSCs diagnoses were collected using the International Classifications of Diseases 10 codes for BCCs, SCCs, and MSCs.

Statistical analysis

Diagnosis data were separated into the number of skin cancers diagnosed each day, month, seasonal quarter, and year from 1983 to 2017. Seasonal quarters were separated into four categories: December through February (winter), March through May (spring), June through August (summer), and September through November (fall). Data were analyzed using chi-square tests, and the data trends of cancer diagnoses over months and years were plotted using Microsoft Excel. The published study Global atlas was constructed using “https://mapchart.net/.”

## Results

**Figure 1:**
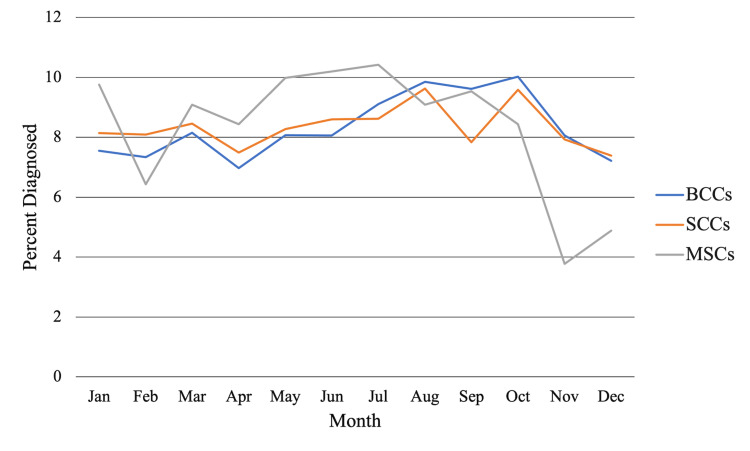
The average percentage of NMSC and MSC diagnoses made each month from 1983 to 2017. BCC: basal cell carcinoma; SCC: squamous cell carcinoma; MSC: melanoma skin cancer; NMSC: non-melanoma skin cancer

**Figure 2:**
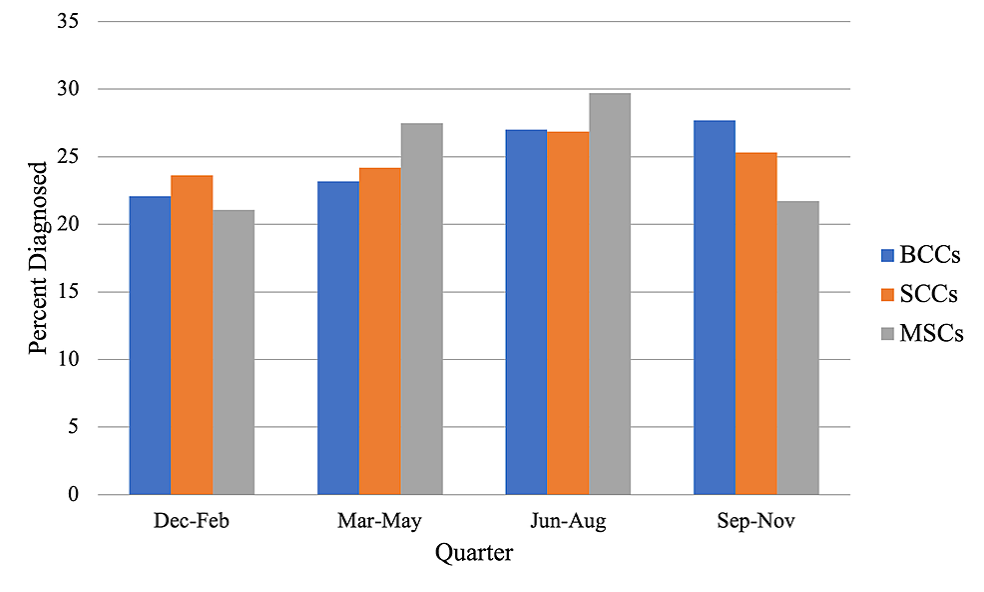
The average percentage of NMSC and MSC diagnoses made each quarter from 1983 to 2017. BCC: basal cell carcinoma; SCC: squamous cell carcinoma; MSC: melanoma skin cancer; NMSC: non-melanoma skin cancer

Chi-square tests showed the highest rates of diagnosis for BCCs in August (9.85%), September (9.62%), and October (10.0%) (Figure 1). For SCCs, the diagnosis rates were the highest in July (8.62%), August (9.63%), and October (9.58%); however, the rates in June (8.60%) were comparable (Figure 1). For MSCs, the diagnosis rates were the highest in May (9.98%), June (10.2%), and July (10.4%), with January (9.76%) trailing close behind (Figure 1). By quarter, the highest diagnosis rate was September through November for BCCs (27.0%, χ^2^ = 76.0, df = 3, p < 0.0001) (Figure 2) and June through August for SCCs (26.9%, χ^2^ = 13.8, df = 3, p = 0.0033) and MSCs (29.7%, χ^2^ = 13.8, df = 3, p = 0.00326) (Figure 2).

**Figure 3:**
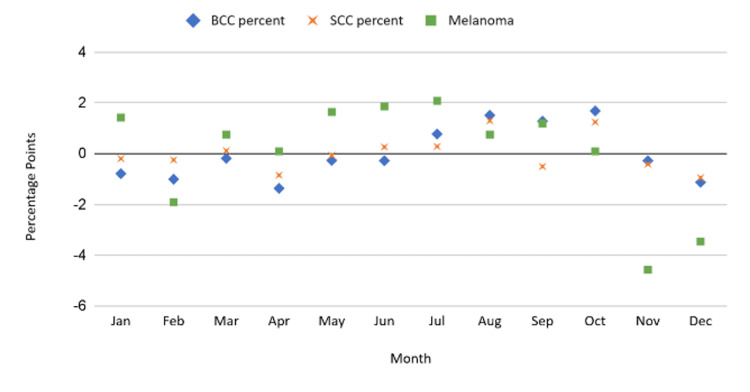
Relative diagnosis rates for SCC, BCC, and MSC subtypes at Physicians East Dermatology Clinic in Greenville, North Carolina, from 1978-2017. BCC: basal cell carcinoma; SCC: squamous cell carcinoma; MSC: melanoma skin cancer; NMSC: non-melanoma skin cancer

**Figure 4:**
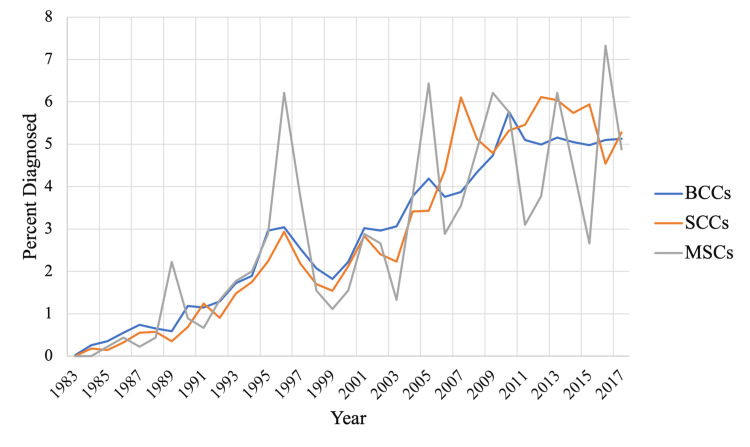
Annual percentage of NMSC and MSC diagnoses made each year relative to the total sum from 1983 to 2017. BCC: basal cell carcinoma; SCC: squamous cell carcinoma; MSC: melanoma skin cancer; NMSC: non-melanoma skin cancer

Analysis of the differences between observed skin cancer diagnoses by month and equal distribution across all months in the event of no seasonality revealed peaks of skin cancer diagnoses corresponding to July through October for BCCs; July, August, and October for SCCs; and May through September for MSCs (Figure 3). Analysis of the patterns of diagnosis of this data over 34 years illustrated a continuously increasing pattern of diagnosis for all three cancer subtypes from 1983 to 2017 (Figure 4).

## Discussion

**Figure 5:**
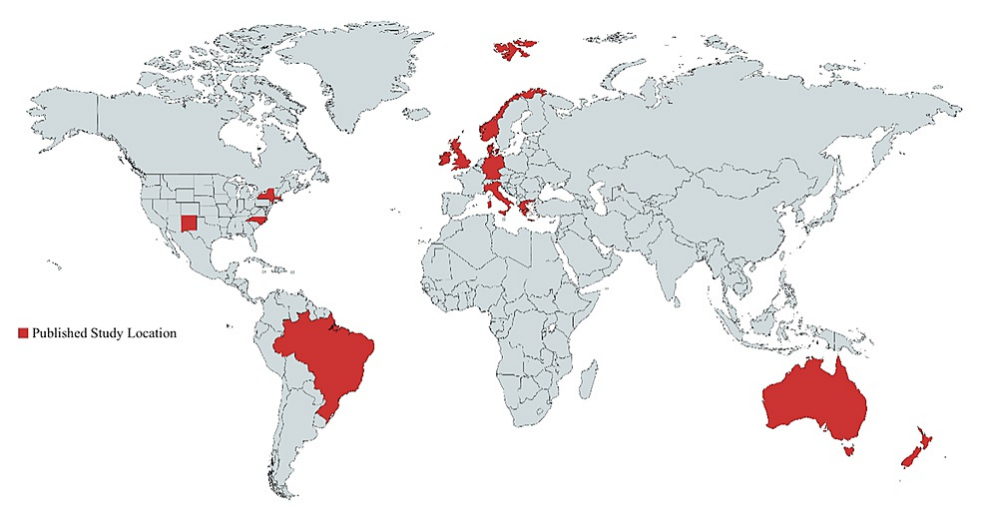
World atlas for locations of published studies on trends in seasonal diagnosis of NMSCs or MSCs. MSC: melanoma skin cancer; NMSC: non-melanoma skin cancer

This study identified a statistically significant pattern of seasonality in both NMSCs and MSCs, which was consistent with the findings of previous studies. The early peak of MSC diagnoses relative to NMSC diagnoses correlated well with the quick progression and presentation of MSCs relative to NMSCs [1-3]. One concern was the potential for continual practice growth over 12 months to conceal trends in seasonality by falsely elevating winter diagnoses. Conversely, the large number of winter holidays in the United States may explain the sharp drop-off in diagnoses of all three cancer subtypes in November and December. A more discrete unit of measurement (e.g., average diagnoses per day) and controlling for office visit chief complaints (e.g., total body skin examination) would assist in delineating the role of these factors. Rommens et al. took a novel quantitative approach based on two months of cumulative preceding UVR exposure, showing a strong linear relationship between UV exposure and skin cancer diagnosis [12]. As a quantitative approach to UVR becomes more predominant, possible geographic confounders affecting UVR exposure will become more critical (e.g., increased geographic altitude limiting the UV blocking effect of ozone, the average summertime temperatures affecting how long people remain outdoors). Increasing the diversity of study locations (Figure 5) will be essential to elucidate the roles of these factors in affecting UVR exposure.

There were several limitations to this study, primarily associated with data availability. The authors did not have access to data regarding BCC, SCC, or MSC subtype diagnoses each month or year; therefore, it was impossible to assess the yearly winter to summer season ratios for NMSCs or MSCs. No data regarding patient demographics (e.g., age, skin type, gender) or skin cancer locations were available. Additionally, cumulative UVR data could not be assessed, as was seen in Rommens et al. [12].

## Conclusions

The seasonality of diagnosis in NMSCs and MSCs is often attributed to healthcare-related factors. High rates in the late summer and fall are thought to be related to delayed cancer presentation. Our data shows a later seasonal BCC peak compared to SCC’s peak, perhaps due to delayed UV effects or healthcare access. Therefore, interventions should be focused on equal year-round access to skin examinations by dermatologists to reduce the influence of healthcare access. Further research should investigate the roles of temperature, quantified UV exposure, and geographic location and their relationships to seasonality.
